# A Case of a Newborn Presenting With a VACTERL-Like Association

**DOI:** 10.7759/cureus.75400

**Published:** 2024-12-09

**Authors:** John Delgado, Logan Atkins, Micah Pippin, Jessan Jishu

**Affiliations:** 1 Family Medicine, Louisiana State University Health Sciences Center, Alexandria, USA; 2 Family Medicine, Rapides Regional Medical Center, Alexandria, USA; 3 Family Medicine, Tulane University School of Medicine, New Orleans, USA

**Keywords:** bicuspid aortic valve disease, congenital birth defect, micropenis, tracheo-esophageal fistula, vacterl association

## Abstract

The VACTERL (vertebral defects, anal atresia, cardiac defects, tracheoesophageal fistula, renal anomalies, and limb abnormalities) association represents an enigmatic syndrome requiring further study. This report describes a full-term neonate born to a multiparous woman who was found, upon further examination, to have multiple congenital abnormalities, including a bicuspid aortic valve, patent foramen ovale, tracheoesophageal fistula (TEF), asymmetric crying facies, microphallus, and a single inguinal testis. The discussion explores environmental and genetic factors that may contribute to this association, as well as similar conditions, such as CHARGE (coloboma, heart defects, choanal atresia, growth retardation, genital abnormalities, and ear abnormalities) syndrome. This study aims to serve as a primer for intellectual inquiry in recognizing and understanding the VACTERL association among healthcare professionals.

## Introduction

The VACTERL association refers to a group of congenital multi-system anomalies that manifest with at least three of the following: vertebral abnormalities (V), anal atresia (A), cardiac malformations (C), tracheoesophageal fistula (TEF), renal dysplasia (R), and limb abnormalities (L) [1]. The VACTERL association is largely a clinical diagnosis, making it possible to misdiagnose patients with other diseases that present similarly, such as CHARGE syndrome, which can involve the following manifestations: coloboma (C), heart defects (H), atresia of choanae (A), restricted growth and mental development (R), genital hypoplasia (G), and ear anomalies (E) [1]. Although these two diseases may be difficult to distinguish in the newborn period, patients with the VACTERL association generally lack the facial features involving the eyes and ears that are typically found in CHARGE syndrome [2]. Nevertheless, many reviews have suggested emphasizing genetic testing, careful neonatal physical exams, and maternal social histories to rule out other conditions [3]. These examinations are critical since the VACTERL association has been associated with several maternal risk factors, including gestational diabetes and chronic obstructive lung disease (COPD) [1]. Indeed, appreciating such risk factors can guide clinicians in accurately diagnosing patients with the VACTERL association. In this case study, we examine a full-term newborn with clinical hallmarks of the VACTERL association.

## Case presentation

Baby D was born at a gestational age of 38 weeks and 5 days, weighing 2.985 kg, with a length of 49 cm and a head circumference of 34.2 cm, to a 27-year-old G3P1A1L1 mother. The mother's blood type was A positive, with negative screens for hepatitis, human immunodeficiency virus (HIV), Group B Streptococcus, and syphilis, and she was immune to Rubella. The mother received routine prenatal care with no abnormalities noted. However, the pregnancy was classified as high-risk due to meconium-stained fluid, active daily smoking, overweight status, and methadone use.

The patient was delivered vaginally in vertex presentation with meconium-stained amniotic fluid, and membranes ruptured during delivery. Apgar scores were eight and nine at one and five minutes, respectively. On initial physical examination, Baby D was placed in a radiant warmer with bubble continuous positive airway pressure (CPAP). Vitals included a temperature of 100.1°F, a heart rate of 166 beats per minute, a respiratory rate of 51 breaths per minute, and a blood pressure of 69/43 mmHg. Head examination revealed asymmetric crying facies on the right with low-set, malformed ears. Genital examination demonstrated impalpable testes and a severe microphallus measuring less than one centimeter.

**Figure 1:**
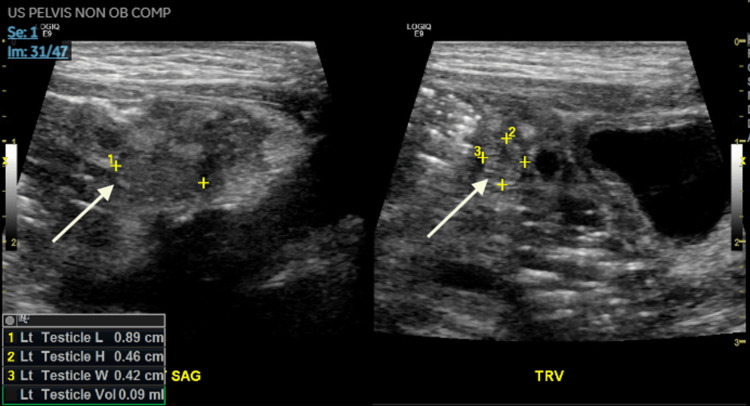
Pelvic ultrasound demonstrating left ovoid structure within the inguinal canal

A pelvic ultrasound was performed to evaluate the impalpable testes, revealing a small ovoid structure in the left inguinal canal with no evidence of a uterus or ovaries (Figure 1).

**Figure 2:**
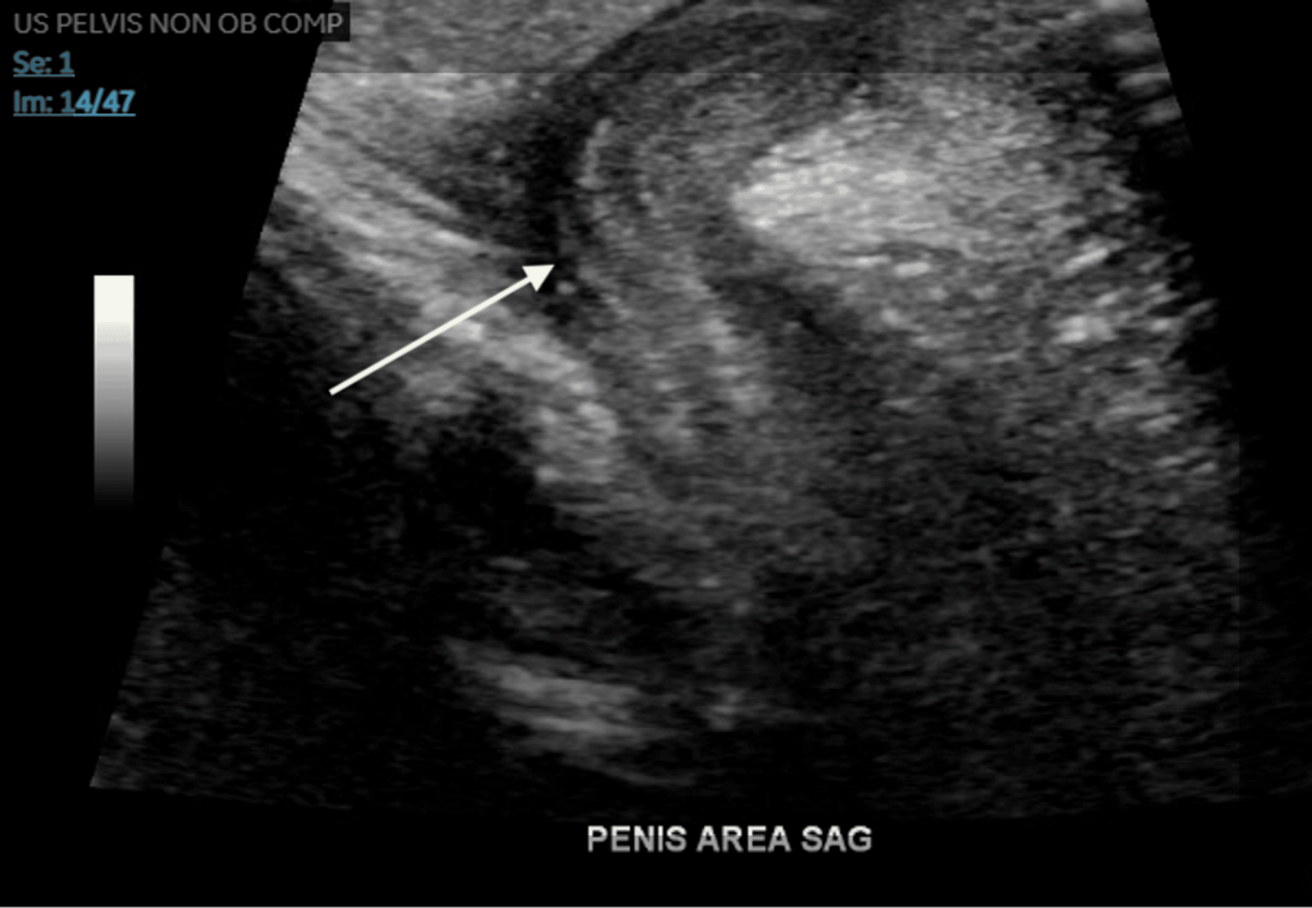
Pelvic ultrasound demonstrating a microphallus

An ultrasound of the pelvis also demonstrated a microphallus (Figure 2).

Baby D was transferred to the neonatal intensive care unit (NICU) for further management and was intubated within three hours of admission due to respiratory distress. The patient was ventilated using conventional methods. An echocardiogram performed within the first 24 hours of life revealed a bicuspid aortic valve, patent foramen ovale, patent ductus arteriosus, and pulmonary hypertension secondary to respiratory distress (Video 1).

**Video 1:** Echocardiogram demonstrating bicuspid aortic valve, patent ductus arteriosus (PDA), and patent foramen ovale (PFO)

**Figure 3:**
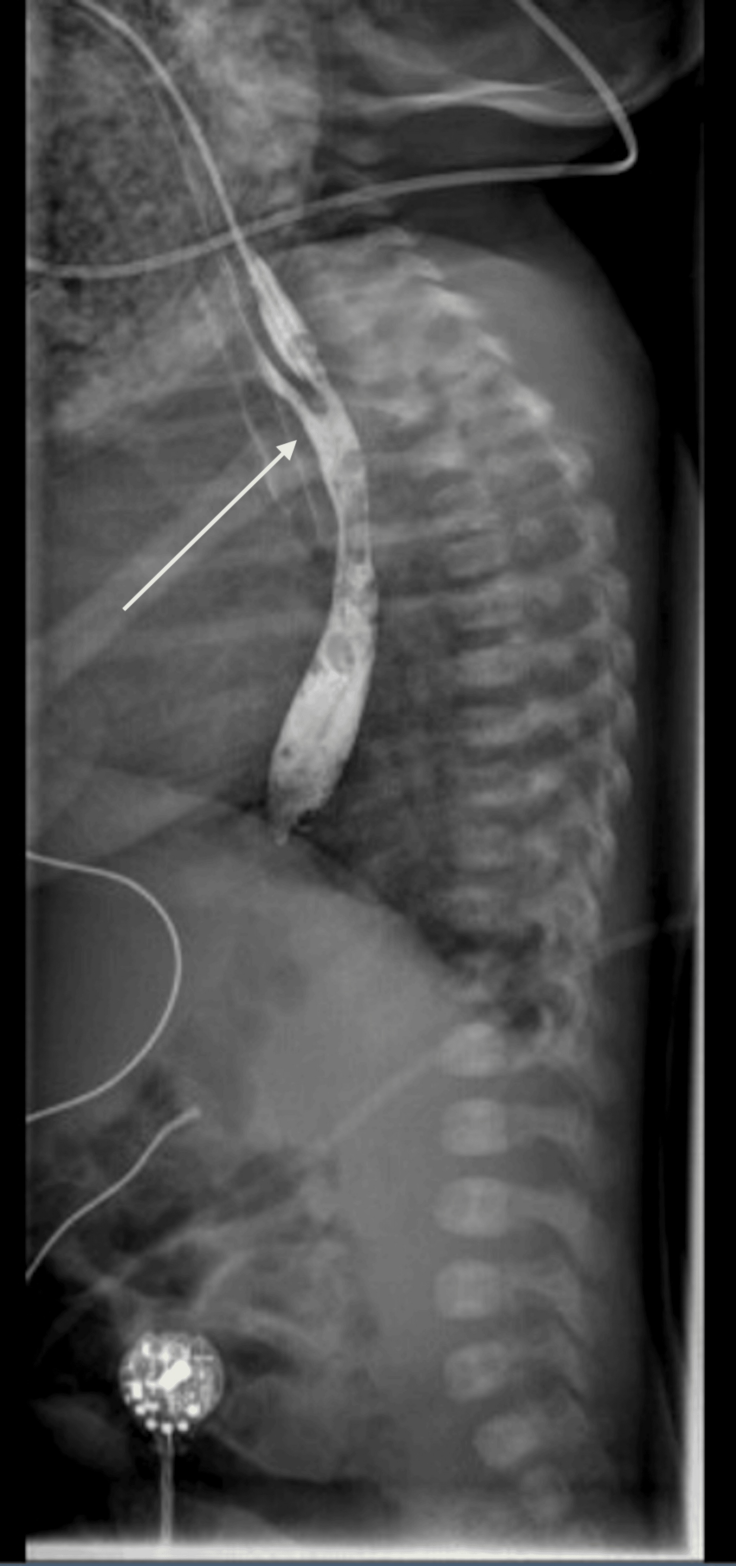
Upper gastrointestinal series demonstrating communicating fistula between esophagus and trachea at approximately level of the second thoracic vertebra

Upon feeding following the echocardiogram, Baby D expressed a large volume of secretions from the trachea and esophagus in addition to a significant volume of air, for which an upper gastrointestinal (GI) series was performed. This demonstrated a TEF at the level of the second thoracic vertebra (Figure 3).

In consideration of these multiple congenital abnormalities, the patient was transferred to a different facility offering a greater degree of care with capabilities for surgical reconstruction indicated for this neonate.

These multiple congenital abnormalities prompted further investigation, for which laboratory and diagnostic findings demonstrated the following results (Table 1).

**Table 1:** Laboratory Analysis WBC: white blood cell, BUN: blood urea nitrogen, TSH: thyroid-stimulating hormone.

Investigation	Patient Value	Reference Range
WBC	30.4 cells/µL	5-19 cells/µL
Hemoglobin	18.9 g/dL	14-19 g/dL
Platelets	239,000 platelets/µL	150-450 platelets/µL
Sodium	139 millimoles/L	135-148 millimoles/L
Potassium	4.9 millimoles/L	3.3-5.1 millimoles/L
Chloride	102 millimoles/L	98-113 millimoles/L
BUN	10 mg/dL	6-19 mg/dL
Creatinine	0.86 mg/dL	0.2-0.4 mg/dL
Glucose	64 mg/dL	50-125 mg/dL
Total bilirubin	4.4 mg/dL	2-6 mg/dL
Direct bilirubin	0.2 mg/dL	0-0.3 mg/dL
Total protein	5.8 g/dL	6-8.4 g/dL
Cortisol	39.7 µg/dL	6.2-19.4 µg/dL (sample 7-10 am)
TSH	18.49 milli-international units/L	2-40 milli-international units/L
Free T4	2.5 ng/dL	2-5 ng/dL

## Discussion

VACTERL associations have a reported incidence of 1/10,000 to 1/40,000 live births, which varies primarily due to the differences in diagnostic criteria used by different governing bodies. There is no prevalence in any geographical areas or ethnic populations for developing VACTERL associations. There is no prevalent sex that is affected; however, some studies suggest males are more commonly affected, although no consensus has been reached at the current time [3]. VACTERL associations are largely sporadic, with some evidence to support genetic markers that can be associated with the presentation of some of the common findings [3,4]. However, genetic counseling should always be considered as it can rule out some disorders that have well-established inheritance patterns [3].

VACTERL associations are heterogeneous disorders characterized by multiple congenital abnormalities affecting different systems; however, in VACTERL-like syndromes, not all abnormalities may be present [1,2]. Additional abnormalities may be observed given the overlap with CHARGE syndrome, trisomy 18, trisomy 21, Fanconi anemia, Townes-Brocks syndrome, and deletion 22q11.2 syndrome, which should be considered in the differential diagnosis [4,5].

Although the exact cause is unknown, it is believed to result from a combination of genetic and environmental factors, which can include mutations of FGF8, FOXF1, HOXD13, LPP, TRAP1, and ZIC3 [1-2]. TNF receptor-associated protein 1 (TRAP1), in particular, encodes a heat-shock protein 90-related mitochondrial chaperone that is involved predominantly in the development of the proximal tubules leading to renal agenesis in patients with autosomal recessive mutations of TRAP1. HOXD13, belonging to the large family of homeobox genes and serving as a downstream target of SHH, plays a significant role in the development of the axial skeleton and limb morphogenesis [1]. Environmental factors such as maternal smoking, primiparity, and elevated BMI, classified as either overweight or obese, have been shown to have an increased incidence of VACTERL association [6,7]. The manifestations of VACTERL associations can vary widely from one individual to another. The diagnosis is made primarily through physical examination, medical imaging, and genetic testing to assess the presence and extent of congenital abnormalities [1].

Laboratory analyses are essential in evaluating any comorbid conditions or factors that could guide the diagnosis of overlapping conditions, such as hematological abnormalities in Fanconi anemia or hypocalcemia in deletion 22q11.2 syndrome [3,4]. Generally, ultrasound sonography can be used as an imaging modality to further investigate for vertebral, anorectal, or renal abnormalities. X-ray, echocardiogram, and contrast studies can be used to investigate limb abnormalities, cardiac malformations, and the presence of TEF, respectively [3,8].

The management of VACTERL associations is mainly surgical and widely complex due to the large array of malformations that can potentially present [9]. Cardiac abnormalities that are incompatible with life would require surgical intervention sooner than malformations of the genitourinary system [3,4,9]. An imperforate anus would require a colostomy with subsequent surgery and re-anastomosis with urological intervention for the presence of any ambiguous genitalia for potential reconstructive surgery [3,4].

TEF is often a part of other constellations of abnormalities, primarily esophageal atresia or cardiac anomalies such as ventricular septal defects, prompting further evaluation if any of these anomalies are found [10]. Treatment of TEF consists of surgical correction performed minimally invasively or through staged procedures depending on the complexity of the defects or comorbid conditions present, such as a right-sided aortic arch, laryngomalacia, or vascular rings [3,4]. Follow-up with a lactation consultant should be considered for appropriate feeding in the future, given the prevalence of feeding difficulties in patients undergoing primary surgical or minimally invasive repair. In the setting of gastroesophageal reflux disease (GERD), neonates will require proton pump inhibitor (PPI) use for the year after repair to prevent possible complications such as Barrett esophagitis, esophageal webs or worsening GERD [2].

Additional complications from the other components of the VACTERL association could include scoliosis and osteoarthritis in the presence of any vertebral abnormalities, thus requiring further surgical intervention, bracing, or physical therapy [7]. Sexual dysfunction, gastrointestinal dysmotility, recurrent urinary tract infections (UTIs), and urinary incontinence can be possible complications in the infant/adolescent with any genitourinary abnormalities diagnosed in the neonatal period, thus requiring further evaluation by specialist care [3].

While the current methods of detecting any antenatal abnormalities, including the vast array of abnormalities that present in VACTERL associations, primarily involve ultrasonographic methods, malformations such as imperforate anus and TEF can go unnoticed unless recognized by the skilled technician and medical interpreter [1,2]. While not present in our patient, polyhydramnios or a single umbilical artery can undoubtedly provide diagnostic clues toward recognizing these VACTERL association abnormalities earlier in the pregnancy [1,2,8]. Thus, sufficient education should be provided to patients about the possibility that some, although not all, elements of the VACTERL association can be definitively diagnosed with absolute certainty [3,4].

## Conclusions

Given its seemingly sporadic nature, the VACTERL association presents a complex syndrome of scientific interest. The findings in the description of this case study regarding a full-term newborn with several hallmarks of the VACTERL association demonstrate the disorder's heterogeneous nature and varying presentations. Identification of newborn findings consistent with components of the VACTERL association should prompt further investigation for coexisting abnormalities. Facilities with adequate specialized care will be paramount in determining the management, prognosis, and quality of life of neonates with the VACTERL association.
